# Vitamin D Levels in Subjects with Prostate Cancer Compared to Age-Matched Controls

**DOI:** 10.1155/2012/524206

**Published:** 2012-12-12

**Authors:** Subhashini Yaturu, Sonya Zdunek, Barbara Youngberg

**Affiliations:** ^1^Department of Endocrinology, Stratton VA Medical Center, Albany, NY 12208, USA; ^2^IT Department, Stratton VA Medical Center, Albany, NY 12208, USA; ^3^Department of Research, Stratton VA Medical Center, Albany, NY 12208, USA

## Abstract

Prostate cancer (PCa) is the second most common cancer in men worldwide and the second leading cause of cancer deaths in men in the United States. Vitamin D is considered to have anticancer properties, currently thought to work mainly through its nuclear receptor or vitamin D receptor. In this retrospective study, we compared vitamin D levels in subjects with PCa with those of age-matched men without PCa. Study subjects included 479 in each group with a mean age of 73 and a mean creatinine of 1.05 and 1.15. Levels of 25 (OH) vitamin D were 28.4 ± 0.54 and 28.05 ± 0.62 in subjects with and without PCa. Levels of 1,25 (OH) vitamin D were 47.2 ± 6.8 and 47.1 ± 7.11 in subjects with and without PCa. In contrast to other studies, we did not find a significant difference in vitamin D levels. Among prostate cancer patients, vitamin D levels correlated positively with age (*r* = 0.12, *P* < 0.02), and were negatively associated with BMI (*r* = −0.13, *P* = 0.003), glucose (*r* = −0.12, *P* < 0.007), HbA1C (*r* = −0.16, *P* = 0.001), and PTH (*r* = −0.21; *P* < 0.0001). The data do not show the causal effect of vitamin D levels on PCa.

## 1. Introduction

Prostate cancer is the most commonly diagnosed cancer in men in the United States [1]. It is estimated that 241,740 men will be diagnosed with and 28,170 men will die of cancer of the prostate in 2012 [1]. Vitamin D from the skin and diet is metabolized in the liver to 25-hydroxyvitamin D (25(OH)D), which is used to determine a patient's vitamin D status [2]. Vitamin D insufficiency affects almost 50% of the population worldwide [2, 3]. In the Third National Health and Nutrition Examination Survey (NHANES III), a cross-sectional multivariate analysis, the lowest quartile of 25(OH)D level (<17.8 ng/mL) was independently associated with all-cause mortality in the general population [4]. Prostate cells contain vitamin D receptors as well as enzymes necessary for vitamin D metabolism. Vitamin D metabolites are considered to have an antiproliferative and a prodifferentiating effect on prostate cancer cell lines in vitro and in vivo. Low levels of plasma vitamin D have been implicated as a possible risk factor for both prostate cancer incidence and advanced disease. In a study to examine the association between vitamin D receptor (VDR) polymorphisms and prostate cancer stage, the authors concluded that low levels of vitamin D may increase the risk of prostate cancer progression [2]. However, meta-analysis of published literature of studies on vitamin D levels and association with prostate cancer reported no association [5, 6] or little evidence to support a major role of vitamin D in preventing prostate cancer or its progression [7]. There are on-going clinical trials to evaluate the benefit of vitamin D in prostate cancer prevention and progression as noted at clinical trials.gov. No studies have compared the vitamin D levels to age-matched controls. The Institute of Medicine's 2011 report on dietary reference intakes for calcium and vitamin D could not make any conclusion regarding whether vitamin D has anticancer activities in humans [4]. Since prostate cancer is common in the elderly and vitamin D deficiency is common in that age group, the association of vitamin D deficiency with prostate cancer may be a simple association with age, not necessarily a factor in the cause or progression of prostate cancer. The aim of this study is to evaluate vitamin D levels in subjects with prostate cancer compared to age-matched controls.

## 2. Materials and Methods

This is a clinical, retrospective study to evaluate vitamin D levels in subjects with or without prostate cancer. This study was approved by the Institutional Review Board as well as Research and Development at Stratton VA Medical Center at Albany, NY, USA. Subjects included patients whose vitamin D levels had been analyzed at Veterans Integrated Service Network (VISN-2) of Veterans Health Administration (VHA) between 2006 and 2009. Patients with end-stage kidney disease and chronic kidney disease, defined as having serum creatinine levels greater than 2.5 mg/dL, were excluded. Using the Current Procedural Terminology (CPT) codes for diagnosis of prostate cancer and the exclusion criteria for the study, 479 subjects with a diagnosis of prostate cancer were identified from review of approximately 5,000 patient records. The dates of diagnosis of prostate cancer were from 1994 to 2011. An additional 479 subjects without diagnoses of prostate cancer were age matched to the prostate cancer group and included in the study. 

The following data was retrieved from the Computerized Patient Record System for all subjects: age, BMI, history of diabetes and hypertension, HbA1C, glucose, creatinine, estimated glomerular filtration rate (eGFR), 25 (OH) vitamin D; 1,25 (OH) vitamin D levels, calcium, and PTH. 

### 2.1. Statistical Analysis

The data is presented as ±SD. A *P* value of <0.05 is considered significant. The difference in vitamin D levels in subjects with prostate cancer compared to age-matched control subjects without prostate cancer was evaluated by the Student's *t*-test (two-sided, heteroscedastic). Correlation of vitamin D levels with other clinical and biochemical parameters was calculated. 

## 3. Results

Study subjects include 479 with prostate cancer and 473 subjects without prostate cancer. Clinical data of subjects with and without prostate cancer is shown in Table 1. The groups are age matched. There is no significant difference in BMI, or in the percent of subjects with hypertension. Biochemical data of subjects with and without prostate cancer is shown in Table 2. There is no significant difference in the vitamin D levels (both 25(OH) vitamin D and 1,25 (OH) vitamin D levels) between the subjects with prostate cancer compared to the age-matched subjects without prostate cancer (*t* = 0.6808; *P* = 0.000595). In the combined analysis of vitamin D in all subjects (with and without prostate cancer), we observed significant negative correlations between vitamin D levels and BMI (*r* = −0.11; *P* < 0.0005) and PTH levels (*r* = −0.26; *P* < 0.0001). In the subgroup analysis of the data of subjects with prostate cancer, we found statistically significant correlations of vitamin D with age (*r* = 0.12; *P* < 0.02), BMI (*r* = −0.13; *P* = 0.003), diagnosis of diabetes (*r* = −0.14; *P* < 0.01), HbA1C (*r* = −0.16; *P* = 0.0008, glucose (*r* = −0.12; *P* < 0.007), and PTH (*r* = −0.21; *P* < 0.0001).

## 4. Discussion

The most important point in the study is that there is no significant difference in the vitamin D levels between the subjects with and without prostate cancer when age matched. These results vary from epidemiological observations that have suggested links between low vitamin D and increased risk of prostate cancer. Calcitriol, the biologically active form of vitamin D (1,25(OH)D), has been shown to be an antiproliferative, prodifferentiation, proapoptotic agent, and an inhibitor of cell migration. Vitamin D deficiency is associated with an increased risk and poor prognosis of several types of cancer. Meta-analysis of genomic studies on the association between the two most studied VDR polymorphisms (FokI and BsmI) have shown significantly higher risk of cancer when the researchers pooled estimates from cancer sites possibly associated with vitamin D levels (prostate, breast, skin, ovary, non-Hodgkin lymphoma) [8]. In the Third National Health and Nutrition Examination Survey (NHANES III, USA), serum 25(OH)D levels less than 17.8 ng/mL were associated with a 26% increased rate of all-cause mortality (mortality rate ratio (95% CI: +8%; +46%)) [4]. 

Our results, showing no correlation between vitamin D levels and prostate cancer, are similar to recent data of some publications. The findings of the NIH epidemiological study noted that this large prospective study did not support the hypothesis that vitamin D is associated with decreased risk of prostate cancer; indeed, higher circulating 25(OH)D concentrations may be associated with increased risk of aggressive disease [9]. In a study from California, Tseng and associates did not find any association of 1,25(OH)_2_ D to prostate cancer [10]. In a case control study from Sweden, there was no association between prostate cancer risk and vitamin D levels [11]. 

Ultraviolet-induced synthesis of vitamin D_3_ in human skin is dependent on several factors, such as time spent out-of-doors, skin pigmentation, and age of subjects, as endogenous skin synthesis capacity decreases with age and sun protection habits, mainly in populations where clothing (garments and veils) covers most of the skin surface. Dietary intake of vitamin D increases serum levels of 25-hydroxyvitamin D [12]. 25(OH)D constitutes the bulk of vitamin D reserves. With supraphysiologic dose administration, large quantities of vitamin D(3) are stored as the native compound, presumably in body fat, and are slowly released to be converted to 25(OH)D [13]. Because of its relatively long half-life (*τ*_1/2_ = 12.9 (SD: 3.6 d)), the serum 25(OH)D level is considered as the best gauge of individual vitamin D status [14]. Elderly people are at high risk for vitamin D deficiency if their life style entails few outdoor activities, their skin is thick, and they exhibit impairment of renal function [15]. 

Our data on all subjects combined is consistent with the results of several publications that the vitamin D levels negatively correlate with BMI [16, 17]. We noted that a lower number of subjects with prostate cancer had diabetes in our study. This is consistent with the epidemiological evidence to show that subjects with diabetes have lower rates of prostate cancer [18].

In the subanalysis of the data from prostate cancer subjects, we noted association of vitamin D levels with glucose and HbA1C levels. Most of the subjects with prostate cancer receive androgen deprivation therapy. Metabolic effects of androgen deprivation therapy for prostate cancer include insulin resistance, diabetes, dyslipidemia, adverse body composition, and metabolic syndrome that contribute to increase in cardiovascular mortality in this population [19, 20]. Kim and associates reported that men with higher HbA1c levels presented with more biologically aggressive prostate tumors at radical prostatectomy [21]. Similar evidence was noted by Hong and associates that although simple history of having DM may not be a significant factor regarding aggressiveness of clinically localized prostate cancer, the glycemic control, as represented by HbA1C level, may be a useful preoperative predictor of aggressive tumor profile among patients with DM who are also diagnosed with clinically localized prostate cancer [22]. 

Loss of bone mineral density is a well-known consequence of androgen deprivation therapy in men with prostate cancer [23, 24]. Review of the results of 12 clinical trials was said to have shown that, at the doses commonly recommended, 500–1,000 mg calcium and 200–500 IU vitamin D per day, men undergoing androgen deprivation lose bone mineral density [23]. 

### 4.1. Drawbacks

This was a retrospective study. Comorbidities were not looked into. In some cases, vitamin D analysis was done prior to the diagnosis of prostate cancer and in others, after the diagnosis of prostate cancer. There was no attempt to account for this or compare results based on when the vitamin D levels were measured.

## 5. Conclusions 

Vitamin D levels are similar in elderly men with or without prostate cancer. 

## Figures and Tables

**Table 1:** Clinical parameters in subjects with prostate cancer compared to age-matched controls without prostate cancer.

Clinical data in subjects with and without prostate cancer			
	P. Ca	No P. Ca	*P* value
Age (years)	73 ± 0.4	74 ± 0.4	0.81
BMI	29.1 ± 0.23	29.3 ± 0.27	0.66
DM (%)	36	42	0.05
HTN (%)	85	86	0.77

P. Ca: prostate cancer; BMI: body mass index; DM: diabetes; HTN: Hypertension.

**Table 2:** Biochemical data in subjects with prostate cancer compared to age matched controls without Prostate cancer.

Biochemical data in subjects with and without prostate cancer			
	P. Ca	No P. Ca	*P* value
Vitamin D (ng/dL)	28.4 ± 0.53	28.1 ± 0.63	0.68
Creatinine	1.15 ± 0.02	1.07 ± 0.02	0.02
eGFR (mL/min)	72 ± 0.86	81 ± 1.3	<0.001
Calcium (mg/dL)	9.5 ± 0.01	9.5 ± 0.02	NS
Glucose (mg/dL)	114 ± 1.6	119 ± 2	0.03
HbA1C	6.4 ± 0.057	6.6 ± 0.07	0.07
PTH (pg/mL)	74 ± 7	81 ± 7	0.53
1,25, (OH) Vit. D (pg/mL)	47 ± 6.8	47 ± 7.1	0.99

P. Ca: prostate cancer; eGFR: calculated glomerular filtration rate; PTH: Parathyroid hormone; 1,25, (OH) Vit. D: 1,25 (OH) vitamin D; *P* < 0.05 is considered significant.
